# EGF stimulates glioblastoma metastasis by induction of matrix metalloproteinase-9 in an EGFR-dependent mechanism

**DOI:** 10.18632/oncotarget.19622

**Published:** 2017-07-27

**Authors:** Xing-Chen Chen, Xiang-Tai Wei, Jun-Hong Guan, Hong Shu, Duo Chen

**Affiliations:** ^1^ Department of Neurosurgery, Shengjing Hospital of China Medical University, Shenyang 110004, P. R. China`

**Keywords:** glioblastoma, epithelial growth factor, epithelial growth factor receptor, signal transducer and activator of transcription 3/5, matrix metalloproteinase-9

## Abstract

Epidermal growth factor (EGF) and EGF receptor (EGFR) play prominent roles in the metastasis of glioblastoma (GBM). However, the molecular mechanisms for the function of EGF and EGFR in GBM metastasis have not been elucidated. Herein, we demonstrate that coactivation of EGF and EGFR drives tumor metastasis in a matrix metalloproteinase-9 (MMP-9)–dependent manner. Expression levels of EGF, EGFR, and MMP-9 were substantially upregulated in the GBM and edema zones of patients, compared with those of paired unaffected participants. Secretion of EGF and MMP-9 was reduced in the cerebrospinal fluid (CSF) after removing GBM for 2 weeks by operation. To the mechanism, MMP-9 was upregulated by activating EGF and EGFR via PI3K/AKT- and ERK1/2-dependent pathways. Moreover, signal transducer and activator of transcription (STAT) 3 and STAT5 mediated the activation of NF-κB by PI3K/AKT and ERK1/2 pathways. This resulted in transactivation of MMP-9 in GBM. Finally, MMP-9 induction facilitated abnormal proliferation, migration, and invasion of cells, which contributed to GBM metastasis.

## INTRODUCTION

Glioblastoma (GBM) is the most common primary brain tumor in adults and poses the highest risk of death of all human malignancies [1]. During metastasis of GBM, the signals of epithelial growth factor (EGF) are amplified by the EGF receptor (EGFR) to promote tumor growth and survival [2]. Elevated concentration of EGFR is accompanied by gene rearrangement to yield EGFR variant III (EGFRvIII), an in-frame deletion of 801 base pairs (bp) of coding sequence from exons 2 to 7 [3]. EGFRvIII is correlated with ligand-independent and constitutive phosphorylation of the receptor [3, 4]. Although tyrosine kinase activity is relatively low for EGFRvIII, [5] this activity is sufficient to hinder interactions with Casitas B-lineage proteins and impede the degradation of EGFR [6]. Detection of the variant length of the receptor is associated with a worse prognosis for glioma patients [7, 8]. The results of a clinical trial of glioma patients [9] confirmed the efficacy of a treatment in which the expression of EGFRvIII was decreased.

Expression of EGFRvIII induces the secretion of interleukin-6 and leukemia inhibitory factor. These cytokines activate gp130 and generate a paracrine loop that promotes the activation of EGFR in neighboring cells [9]. The phosphorylated forms of EGFR and EGFRvIII also may interact directly [10]. EGFR can function as a kinase, phosphorylating EGFRvIII and driving the progression of GBM in a signal transducer and activator of transcription (STAT) 3- and STAT5-dependent mechanism [11]. These observations support an initiating role of EGFR in GBM, although EGFRvIII mediates ligand-independent phosphorylation of the receptor [3, 4].

The STAT protein family comprises intracellular transcription factors that may play pro-oncogenic roles in a tumor environment. STAT3 induces transformation of glial cells when coexpressed with EGFRvIII and forms a complex with EGFRvIII in the nucleus [12]. STAT5β also may complex with EGFRvIII and contribute to survival of GBM cells [13]. STAT5 was found to regulate glioma cell invasion [14]. Specifically, inhibition of STAT5β induces G1 cell cycle arrest, which reduces tumor invasion in human GBM multiforme cells [15]. However, the roles of STAT3 and STAT5 in the metastasis of GBM are complicated and not fully understood.

In a comparative analysis, matrix metalloproteinase-9 (MMP-9) was identified as the only significant prognostic factor for GBM [16]. Low expression of MMP-9 is correlated with better survival outcomes, [16] and MMP-9 is upregulated in human GBM [17]. MMP-9 is a candidate biomarker for high-grade glioma [18] that directs the migration and invasion of serum-stimulated GBM [19]. In GBM cells treated with micro (mi) RNA-181c, EGFR activates MMP-9 by inducing the phosphorylation of AKT [20].

We sought to elucidate the mechanisms by which EGF regulates the expression and activity of MMP-9, thereby contributing to GBM metastasis. We found that expression levels of EGF, EGFR, and MMP-9 were elevated during the course of GBM progression, but were downregulated 2 weeks postoperatively, especially in the cerebrospinal fluid (CSF). Herein, we demonstrate that EGF regulates the expression of MMP-9 via EGFR in PI3K/AKT-dependent and ERK1/2-dependent STAT3 and STAT5 mechanisms. NF-κB, which is downstream of STAT3 and STAT5, transcriptionally regulates the expression of MMP-9 and contributes to the proliferation, migration, invasion, and metastasis of GBM.

## RESULTS

### The expression and activity of MMP-9 were elevated in GBMs and were alleviated by operation

**Figure 1:**
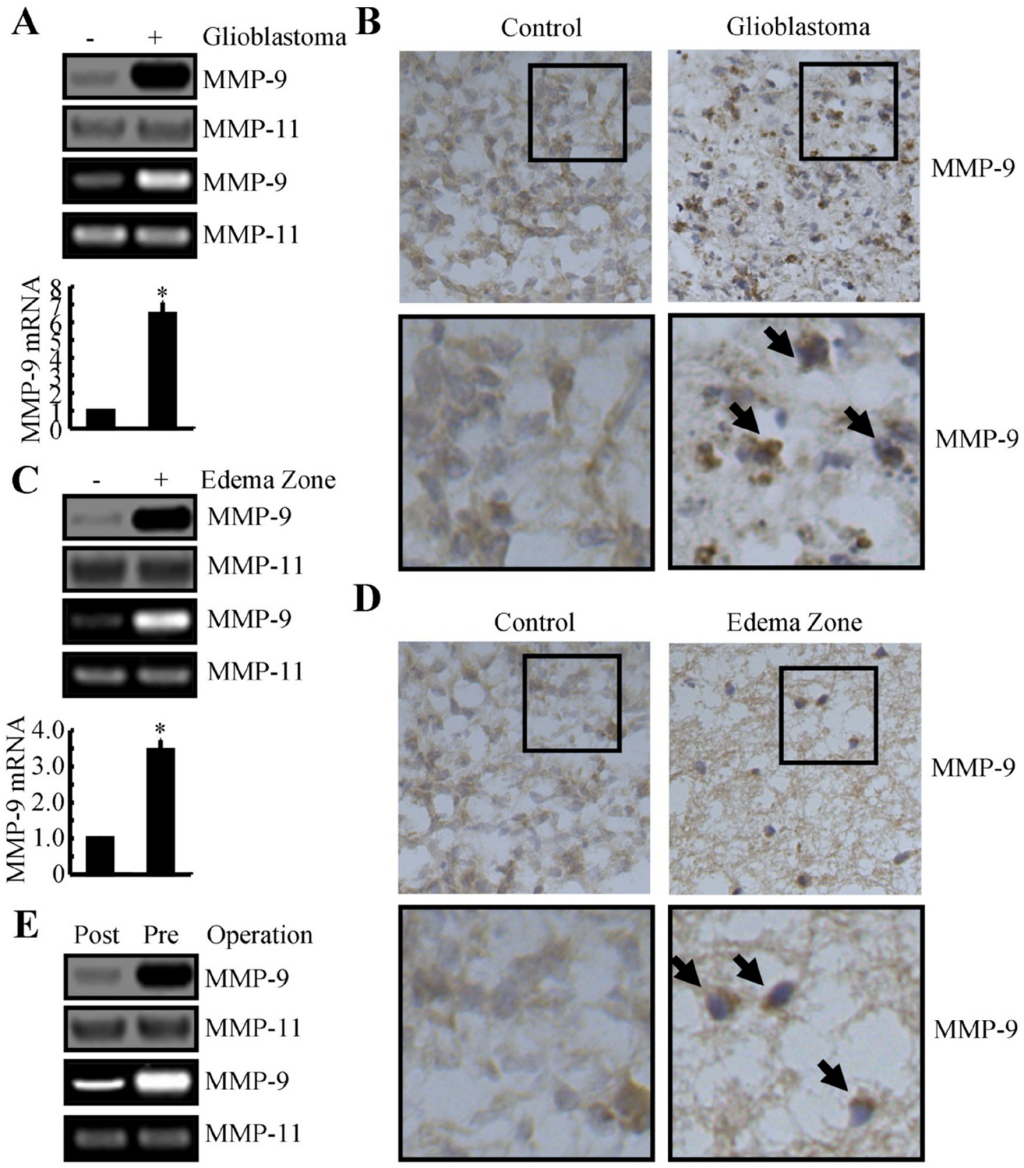
MMP-9 expression is increased in human GBM tissues and edema zones, relative to normal controls **(A-D)** GBM tissues, edema zones, and paired normal tissues were collected from the Shengjing Hospital of China Medical University (n=3). Total mRNA was isolated with phenol-chloroform, and total protein was extracted with RIPA buffer. (A, C) The expression levels of MMP-9 mRNA and protein and the activity of MMP-9 were determined by qRT-PCR, Western blotting, and zymography, respectively. GAPDH and MMP-11 were run as internal controls. (B, D) The morphology of MMP-9 was determined by immunohistochemistry. **(E)** CSF was collected from patients with GBM preoperatively and 2 weeks postoperatively and assessed for MMP-9 protein expression and activity, with MMP-11 included as an internal control. Data represent the mean ± SE of at least 3 independent experiments. *, *P* < 0.05 versus paired normal controls or preoperative groups.

MMP-9 has been found to be elevated in affected tissues of patients with GBM, compared to the tissues of healthy individuals [17]. We analyzed specimens from the edema zone and GBM region and compared our findings to those of paired normal tissues. The results of Western blotting, zymography, and qRT-PCR indicated that expression and activity of MMP-9 were markedly elevated in GBMs compared to paired normal tissues (Figure 1A). Immunostaining experiments were carried out to determine the morphology and expression levels of MMP-9 in GBMs. Compared to normal controls, MMP-9 was highly increased in GBMs (Figure 1B).

MMP-9 is a form of gelatinase that can be secreted from GBM tumors to the edema zone, where it degrades the extracellular matrix [20]. The activity of MMP-9 facilitates the migration of tumor cells from the original site and induces inflammation [21]. We evaluated MMP-9 in the edema zone and found that the expression and activity of MMP-9 were substantially elevated (Figure 1C, 1D). We detected MMP-9 in the CSF preoperatively (Figure 1E) and found that MMP-9 was substantially decreased in the CSF postoperatively (Figure 1E). Hence, surgical resection of GBM is efficacious, and MMP-9 has utility as a biomarker for GBM.

### EGF and EGFR were induced during GBM progression and development

**Figure 2:**
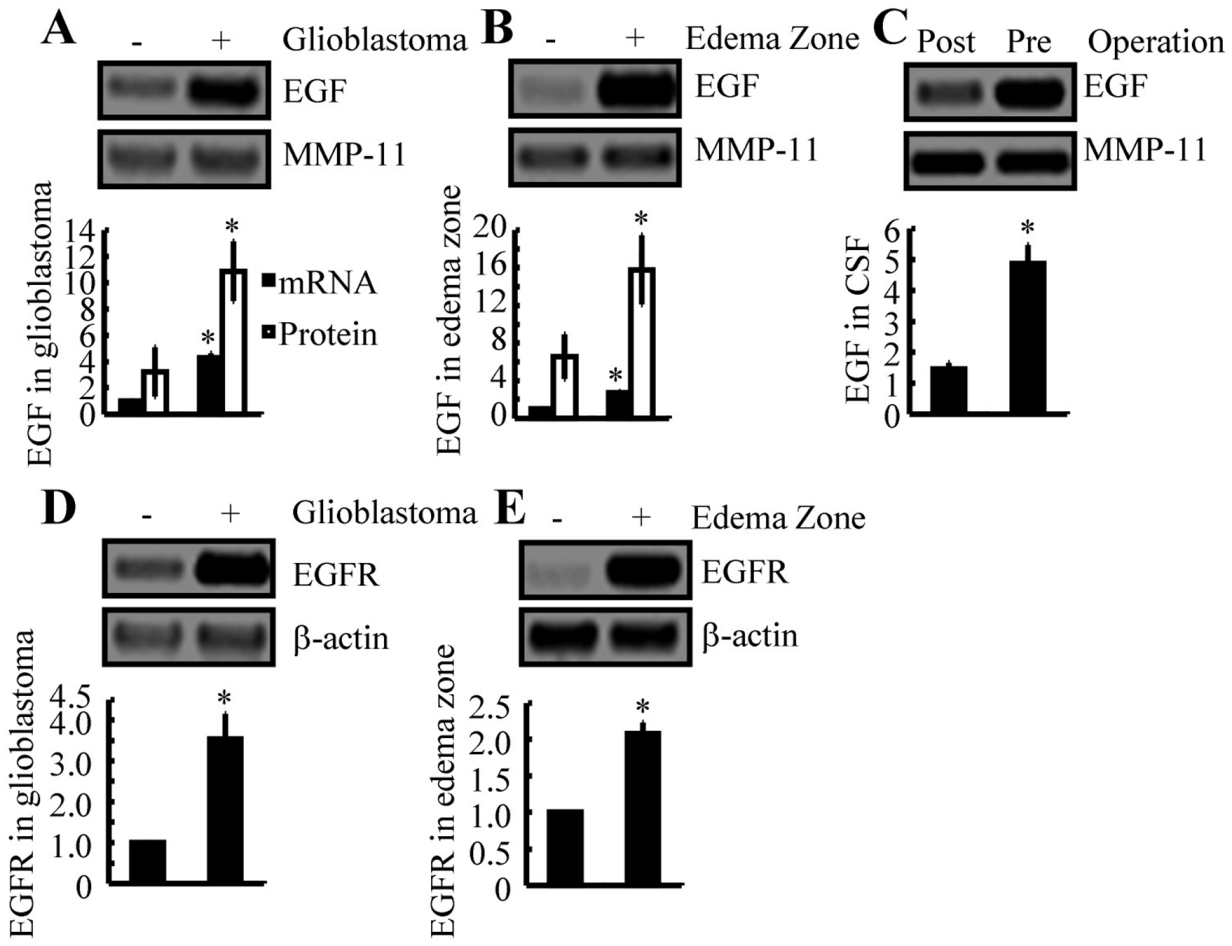
EGF and EGFR expression are increased in human GBM tissues and in the edema zone, relative to normal controls **(A, B, D, E)** GBM tissues, the edema zone, and paired normal tissues were collected from Shengjing Hospital of China Medical University (n=3). Total mRNA was isolated with phenol, and total protein was extracted with RIPA buffer. The mRNA and protein expression levels of EGF (A, B) or EGFR (D, E) were determined by qRT-PCR and Western blotting, respectively. GAPDH and β-actin were included as internal controls. **(C)** CSF was collected from patients with GBM preoperatively and 2 weeks postoperatively. The protein expression of EGF was determined by Western blotting, with MMP-11 as an internal control. Data represent the mean ± SE of at least 3 independent experiments. *, *P* < 0.05, versus paired normal controls or preoperative groups.

We next aimed to delineate the signaling cascade of MMP-9 induction in GBM. Other investigators have shown that EGF and EGFR signaling cascades are critical for the metastasis of GBMs [22]. We determined that EGF mRNA and protein expression levels were substantially upregulated in GBM tumor regions and edema zones (Figure 2A, 2B). Postoperatively, EGF production was markedly reduced in the CSF (Figure 2C). The expression of EGFR also was induced in the GBM and edema zones of tumors (Figure 2D, 2E). These observations support the hypothesis that the EGF and EGFR signaling pathways might be correlated to regulation of MMP-9 activity, which in turn promotes metastasis of GBM.

### EGFR amplifies EGF signals to activate MMP-9 and the ERK1/2, PI3K/AKT, STAT3, and STAT5 pathways

**Figure 3:**
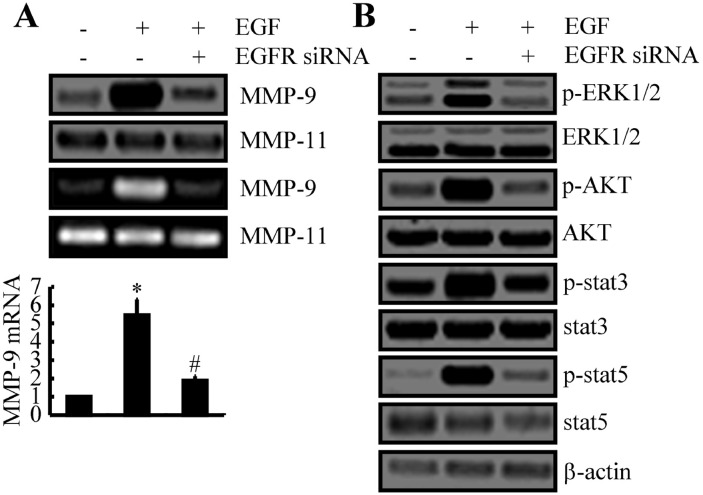
EGF upregulates the expression of MMP-9 and the activities of ERK1/2, AKT, STAT3, and STAT5 pathways via EGFR **(A, B)** A172 cells were treated with EGF (10 ng/ml) in the absence or presence of EGFR siRNA for 48 h. Total mRNA, total protein, and conditioned medium were collected. (A) Expression levels of mRNA and protein and the activity of MMP-9 were determined by qRT-PCR, Western blotting, and zymography, respectively. GAPDH and MMP-11 served as internal controls. (B) Phosphorylation of ERK1/2, AKT, STAT3, and STAT5 were determined by Western blotting. Data represent the mean ± SE of at least 3 independent experiments. *, *P* < 0.05 versus vehicle-treated controls; #, *P* < 0.05 versus cells treated with EGF alone.

When transfected into A172 cells, siRNA that targeted EGFR blocked the capacity of EGF to stimulate the expression and activity of MMP-9 (Figure 3A). EGF treatment stimulated the phosphorylation of factors in related signaling pathways, including ERK1/2, AKT, STAT3, and STAT5 (Figure 3B), and EGFR siRNA similarly blocked these effects (Figure 3B). Hence, these signaling molecules likely are regulators of the expression and activity of MMP-9.

**Figure 4:**
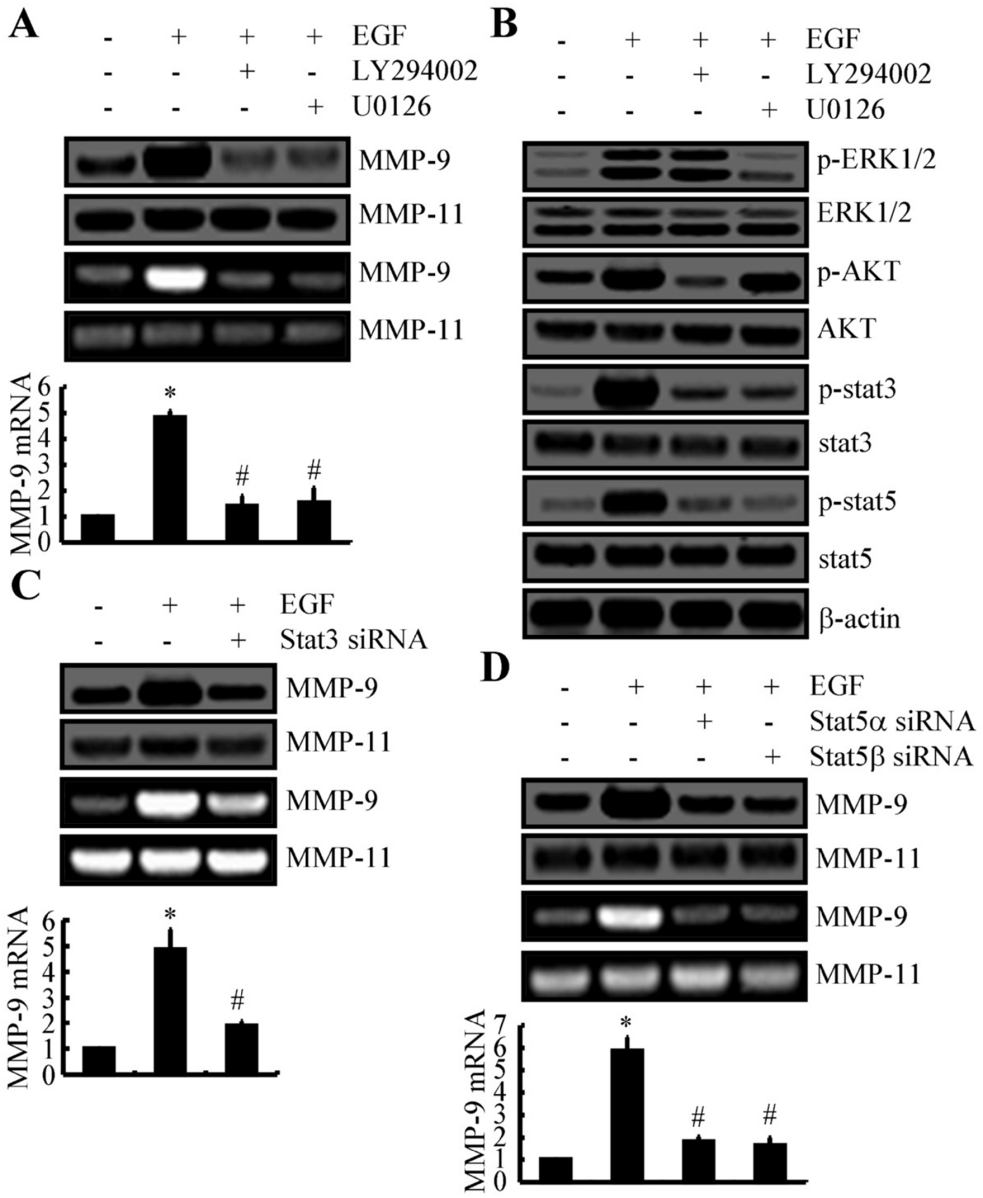
ERK1/2, PI3K/AKT, STAT3, and STAT5 pathways mediate the stimulatory effects of EGF on the expression and activity of MMP-9 in A172 cells **(A, B)** A172 cells were treated with EGF (10 ng/ml) in the presence or absence of LY294002 (10 μM) or U0126 (10 μM) for 48 h. (A) Expression levels of mRNA and protein and the activity of MMP-9 were determined by qRT-PCR, Western blotting, and zymography, respectively. GAPDH and MMP-11 served as internal controls. (B) Phosphorylation of ERK1/2, AKT, STAT3, and STAT5 were determined by Western blotting. **(C, D)** In some experiments, A172 cells were treated with EGF (10 ng/ml) in the presence or absence of STAT3 or STAT5 siRNA. The expression levels of mRNA and protein and the activity of MMP-9 then were determined. Data represent the mean ± SE of at least 3 independent experiments. *, *P* < 0.05 versus vehicle-treated or vector-transfected controls; #, *P* < 0.05 versus cells treated with EGF.

A172 cells subsequently were exposed to inhibitors of PI3K/AKT and ERK1/2 (LY294002 and U0126, respectively) in the presence of EGF. These inhibitors downregulated the expression and activity of MMP-9 under these conditions (Figure 4A). Exposure to LY294002 and U0126 also inhibited the phosphorylation of STAT3 and STAT5 (Figure 4B). For A172 cells that were transfected with STAT3 siRNA, the capacity of EGF to stimulate the expression and activity of MMP-9 was significantly impaired (Figure 4C). Similarly, knockdown of STAT5α and STAT5β attenuated these effects of EGF (Figure 4D). Therefore, ERK1/2, PI3K/AKT, STAT3, and STAT5 upregulate the expression and activity of MMP-9 by mediating upstream EGF.

### The p65 subunit of NF-κB transcriptionally activates MMP-9 in a STAT3- and STAT5-dependent mechanism

**Figure 5:**
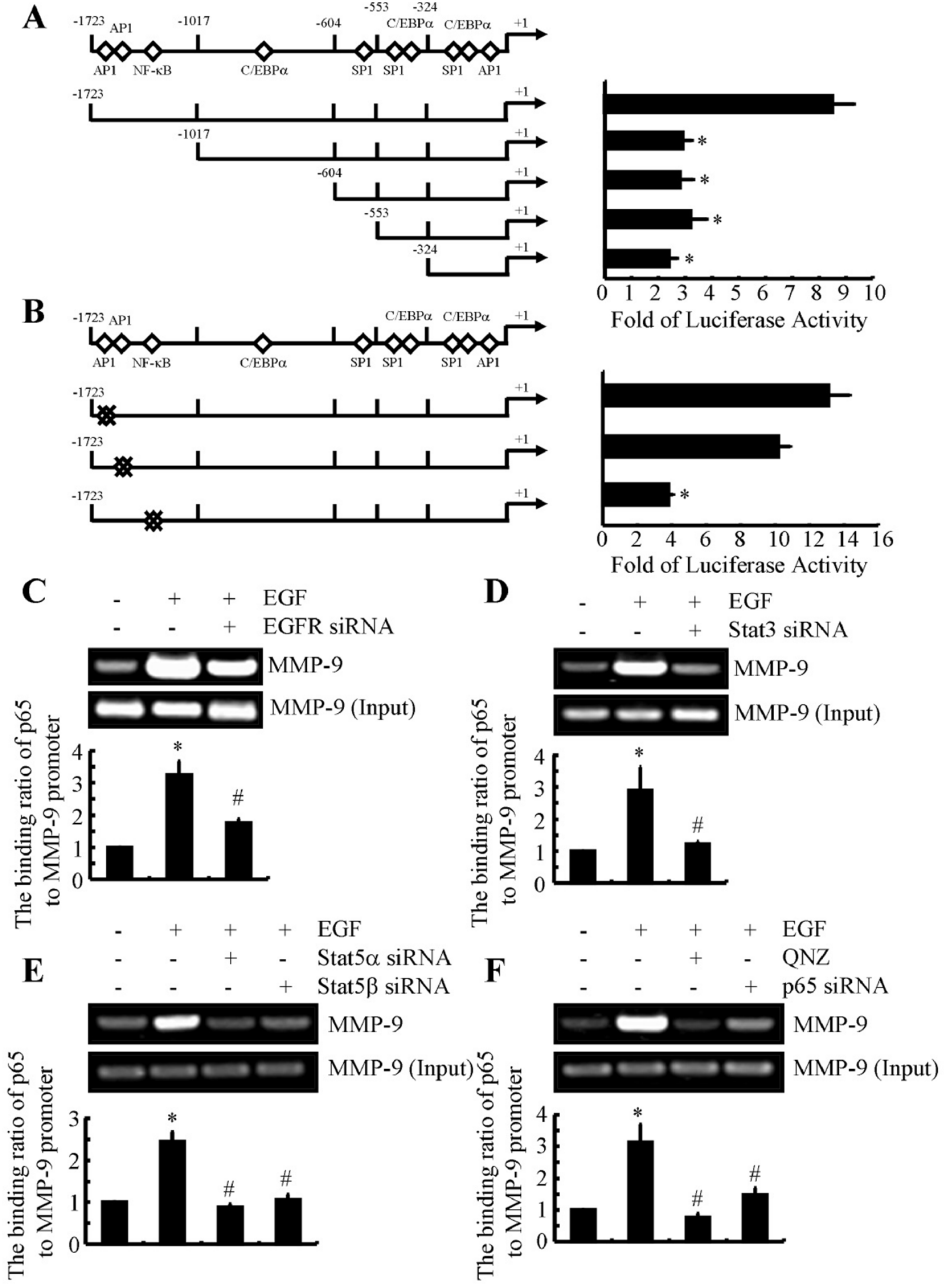
EGF induces binding of the p65 subunit of NF-κB p65 to the *MMP-9* promoter in human GBM cells **(A)** A172 cells transfected with truncated fragments of the *MMP-9* promoter were treated with EGF. **(B)** In some experiments, A172 cells transfected with mutated AP1 or NF-κB of the *MMP-9* promoter were incubated with EGF for 48 h. The luciferase activity of the *MMP-9* promoter was determined with a luciferase detection system. **(C-F)** A172 cells were treated with EGF (10 ng/ml) in the presence or absence of siRNAs targeted to EGFR, STAT3, STAT5, or p65, or were treated with QNZ (1 μM). The binding activity of the p65 subunit of NF-κB to the *MMP-9* promoter was determined by ChIP. Data represent the mean ±SE of at least 3 independent experiments. *, *P* < 0.05 versus vehicle-treated or vector-transfected controls; #, *P* < 0.05 versus cells treated with EGF alone.

Several authors have demonstrated that MMP-9 expression is regulated by NF-κB in airway epithelial cells [23]. We sought to determine whether EGF-induced MMP-9 expression also is dependent on NF-κB. Specifically, we conducted promoter assays to identify binding sites for NF-κB in the *MMP-9* promoter that regulate EGF-induced expression of MMP-9. Before EGF exposure, A172 cells were transiently transfected with a construct encompassing the 5′-flanking region of the human *MMP-9* gene from -1723 to -6 bp (-1723/-6) (Figure 5A). Subsequent EGF treatment increased activity of the *MMP-9* promoter by more than 8-fold (Figure 5A). Deletion of the region from -1723 to -1017 bp (-1017/-6) suppressed luciferase activity of the *MMP-9* promoter; however, additional sequence deletion did not further alter luciferase activity (Figure 5A). The results of bioinformatic analysis of the consensus sequence (-1723/-1017) indicated the presence of an NF-κB binding site, which could be responsible for EGF-induced MMP-9 synthesis. Insertion of a point mutation in this NF-κB site substantially decreased EGF-induced luciferase activity, relative to the wild-type promoter (Figure 5B).

We conducted ChIP to examine the binding of NF-κB to its putative sites on the *MMP-9* promoter. As shown in Figure 5C, binding of the NF-κB p65 subunit to the *MMP-9* promoter markedly increased following exposure of A172 cells to EGF. This binding was reduced in A172 cells that had been transfected with EGFR siRNA (Figure 5C). Similarly, cells transfected with STAT3 or STAT5 siRNAs exhibited reduced EGF-induced binding of the NF-κB p65 subunit to the *MMP-9* promoter (Figure 5D, 5E). This reduced binding also was observed when cells were exposed to QNZ, a p65 inhibitor (Figure 5F). To exclude nonspecific effects of the inhibitor, we transfected A172 cells with p65 siRNA. Under these conditions, binding of NF-κB to the *MMP-9* promoter was decreased (Figure 5F). Collectively, these results indicate that the p65 subunit of NF-κB is essential for EGF-stimulated expression of MMP-9.

### EGF-induced MMP-9 promotes migration and invasion of GBM cells

**Figure 6:**
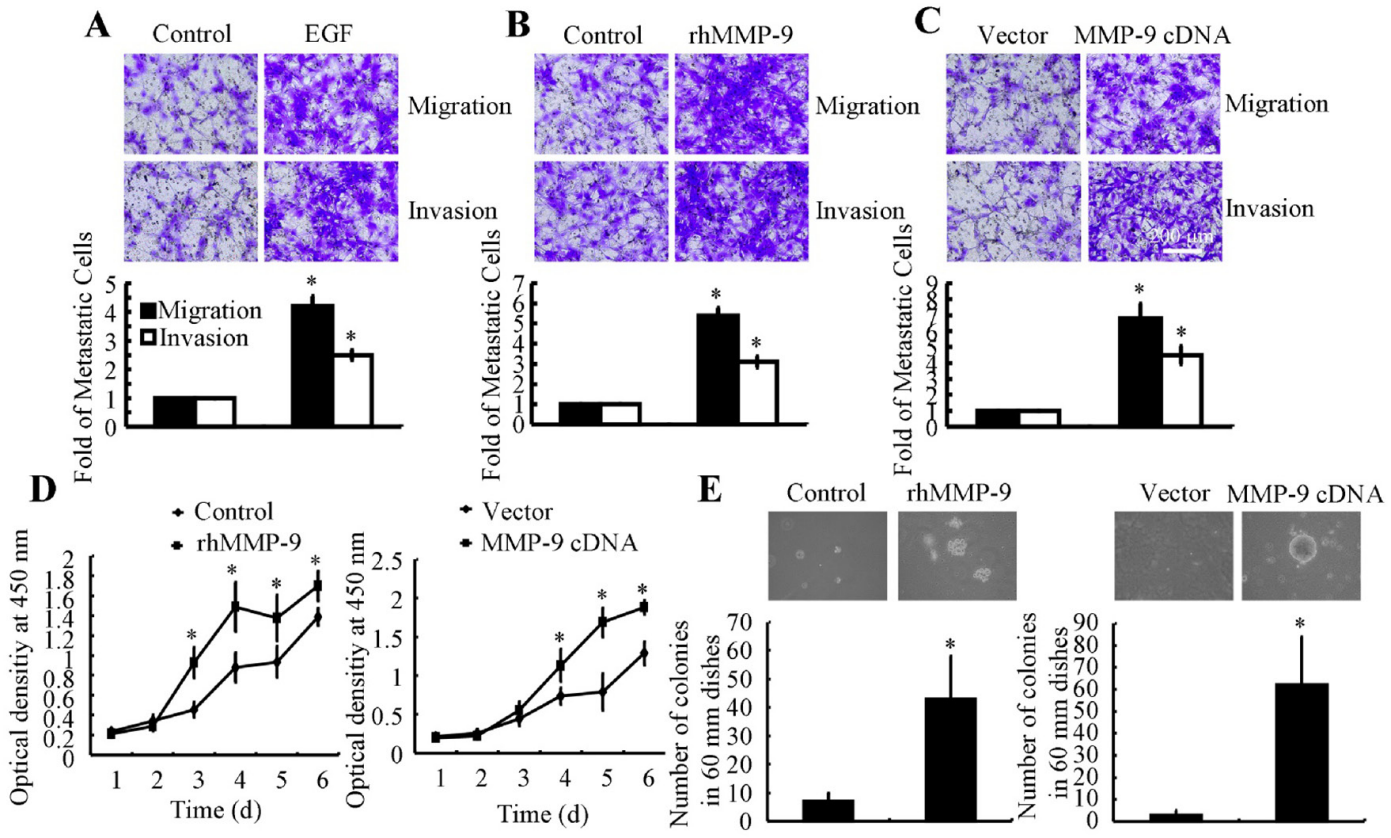
MMP-9 overexpression increases tumor migration, invasion, and abnormal proliferation A172 cells were treated with EGF (10 ng/ml) or rhMMP-7 (1 ng/ml) in serum-free DMEM or in 0.3% soft agar medium. In some experiments, cells were transfected with MMP-9 cDNA or empty vector before applying serum-free medium or seeding in 0.3% soft agar medium. **(A-C)** The migration and invasion of A172 cells were determined with Transwell inserts. **(D)** The proliferation rate was determined with a cell counting kit. **(E)** The number of colonies in 60-mm dishes was counted. Representative images are depicted. Data represent the mean ± SE of at least 3 independent experiments. *, *P* < 0.05 versus vehicle-treated or vector-transfected controls.

Given that EGF induces MMP-9 activation, we examined the effects of EGF and MMP-9 on migration and invasion of A172 cells. EGF treatment augmented cell migration and invasion (Figure 6A). Exposure to recombinant human (rh) MMP-9, a downstream target of EGF, also stimulated cell migration and invasion (Figure 6B). MMP-9 overexpression-induced by transfection with a plasmid containing MMP-9 cDNA-also enhanced the migration and invasion of A172 cells (Figure 6C).

### MMP-9 in abnormal proliferation of GBM cells

MMP-9 correlates with the degree of malignancy in human GBMs [18]. Therefore, we examined the relationship between MMP-9 and GBM proliferation. As shown in Figure 6D, treatment with rhMMP-9 (100 ng/ml) or transfection with MMP-9 cDNA increased the proliferation rate of A172 cells in serum-deprived DMEM. A172 cells also were seeded in 0.3% soft agar medium to determine whether MMP-9 can stimulate anchorage-independent growth, a hallmark of the epithelial-to-mesenchymal transition. We found that rhMMP-9 or MMP-9 cDNA significantly increased the number of colonies in soft agar medium (Figure 6E). Hence, MMP-9 directs abnormal proliferation of GBM cells.

### MMP-9 is responsible for GBM metastasis

**Figure 7:**
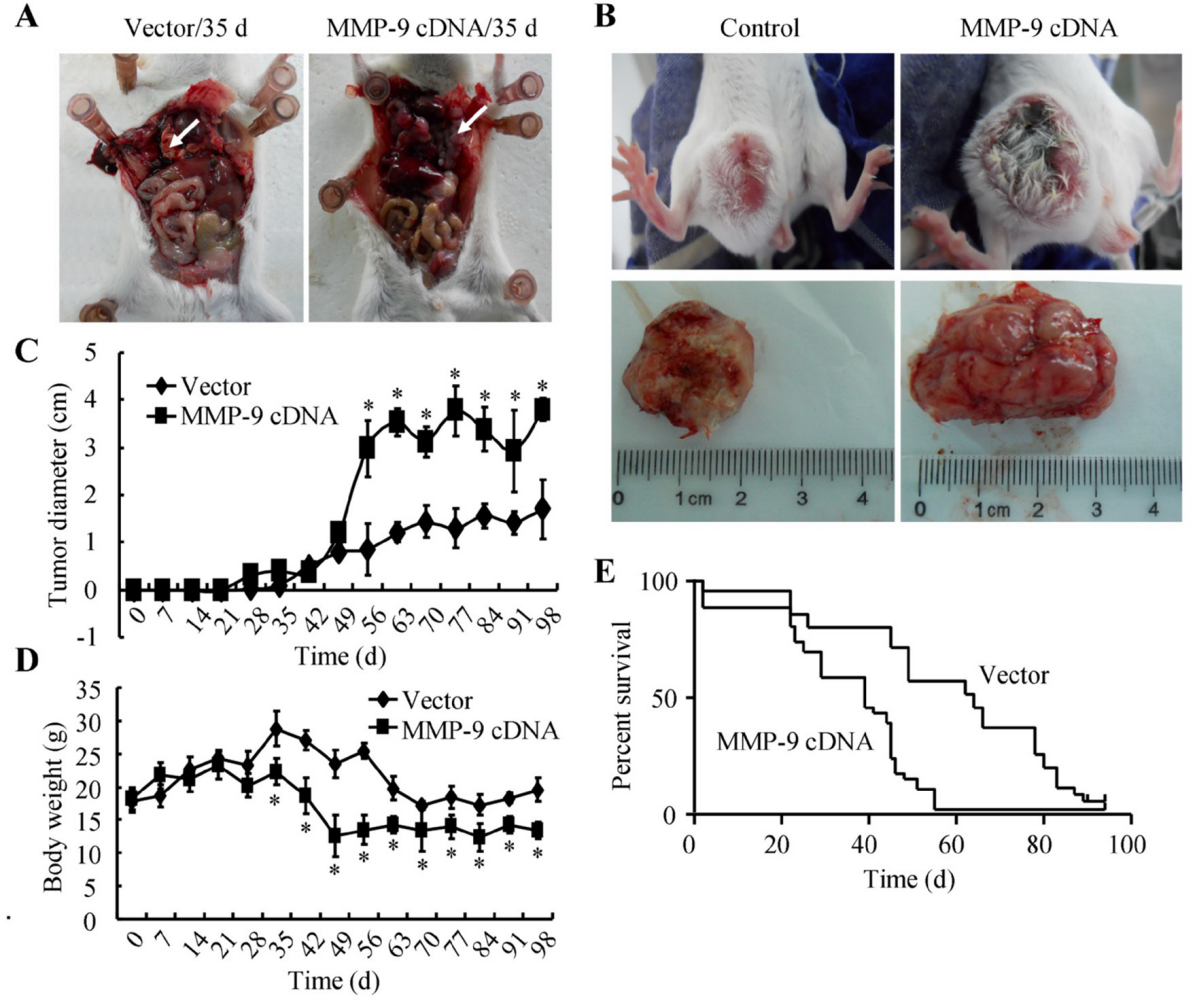
MMP-9 plays critical roles in triggering the metastasis of GBM **(A, D, E)** Mice were injected via the tail vein with A172 cells that had been transfected with MMP-9 cDNA or empty vector (n=15). Injections were carried out weekly for 5 weeks. (A) Metastatic tumors were observed for animals that underwent tail-vein injection of A172 cells overexpressing MMP-9 but not for animals injected with cells carrying the empty vector. Body weight (D) and survival rate (E) were determined, and the tumor diameter **(B, C)** was assessed periodically. *, *P* < 0.05 versus the vector-transfected controls.

To confirm our *in vitro* observations, mice were injected with A172 cells transfected with MMP-9 cDNA or empty vector. Ectopic expression of MMP-9 increased lung colonization *in vivo*, yielding more micrometastases (Figure 7A). Cells transfected with MMP-9 cDNA or empty vector also were injected subcutaneously into mice. The results of daily measurement of tumor diameters indicated that MMP-9 overexpression increased tumor size (Figure 7B, 7C). Moreover, transfection with MMP-9 cDNA decreased the average body weight of mice (Figure 7D) and increased the mortality rate (Figure 7E). Therefore, under conditions of MMP-9 overexpression, malignant transformation is enhanced.

## DISCUSSION

**Figure 8:**
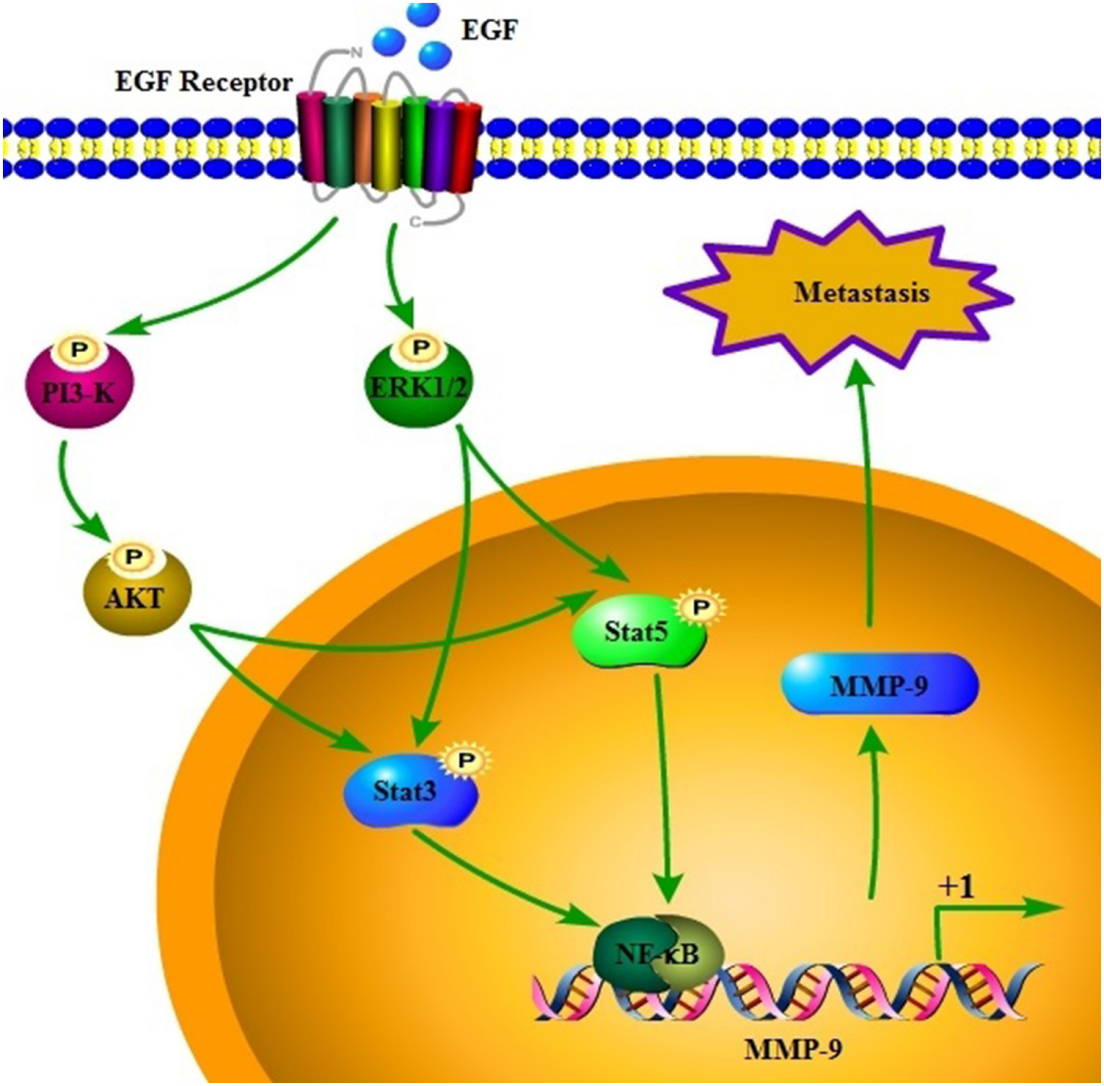
Signaling cascades regulate MMP-9 in response to EGF, and MMP-9 mediates GBM metastasis EGF induces the expression and activity of MMP-9 via EGFR in human GBM cells. ERK1/2, PI3K/AKT, STAT3, STAT5, and NF-κB pathways mediate EGF and EGFR signals to regulate MMP-9 expression. Transactivation of MMP-9ultimately mediates abnormal proliferation, migration, and invasion of cells in GBM, which contributes to metastasis.

MMP-9 upregulation has been detected in GBMs, [16] and MMP-9 is involved in GBM metastasis [19]. To extend these findings, we dissected the signaling pathways responsible for MMP-9 regulation in EGF-stimulated A172 cells. We found that EGF induces the expression and activity of MMP-9 in an EGFR-dependent mechanism. ERK1/2, PI3K/AKT, STAT3, and STAT5 are crucial mediators of EGF, which in turn activates MMP-9 in an NF-κB–dependent mechanism. Upregulation of MMP-9 augments the abnormal proliferation, migration, and invasion of GBM cells, thereby contributing to tumor metastasis in the mouse lung (Figure 8).

Under normal physiological conditions, MMP-9 is strictly maintained at basal levels. In humans, MMP-9 is upregulated in GBM, [17] and MMP-9 is a candidate biomarker for high-grade glioma [18]. Other authors have demonstrated that MMP-9 coordinates the migration and invasion of serum-stimulated GBM [19]. In multiple studies, the essential roles of MMP-9 in GBM migration and invasion have been delineated [24, 25]. In agreement with previous findings, we determined that MMP-9 expression and activity were highly upregulated in GBM cells compared to paired controls.

EGFR is overexpressed in approximately 60% to 90% of all GBMs, and the extent of EGFR overexpression is correlated with poor patient outcomes [8, 26]. Upregulation of the gene encoding EGFR is responsible for aberrant EGFR expression in 30% to 40% of primary brain tumors or brain tumor–derived cell lines [27, 28]. In the remaining cases, the mechanisms must involve excessive and sustained EGFR signaling owing to increased EGFR protein levels. We determined that EGFR mediates the stimulatory effects of EGF on the expression and activity of MMP-9. Consistent with our results, other investigators have demonstrated that EGF activates MMP-9 in the brain [29]. EGFR functions downstream of EGF and stimulates the migration and invasion of human ameloblastoma in an MMP-9 dependent mechanism [30]. EGFR signaling also is necessary for MMP-9 activation in GBM [20]. Therefore, EGF and EGFR signaling pathways are critical for MMP-9 upregulation in GBM.

Intracellular signaling to transduce the EGF stimulus may involve activation of ERK1/2 and PI3K/AKT pathways. Wang *et al.* [20] reported that miRNA-181c inhibits EGFR-dependent activation of MMP-9 by suppressing AKT phosphorylation in GBM. Although further verification is needed, EGF and EGFR signals may stimulate migration and invasion of MDA-MB-231 cells in PI3K- and AKT-dependent pathways [31, 32]. EGF also promotes invasion of vestibular schwannoma tumors via ERK1/2 pathways [33]. EGF and EGFR signaling pathways are crucial for activation of ERK1/2 pathways [11], which in turn are responsible for the expression and activation of MMP-9 [34]. Furthermore, MMP-9 regulates migration of hepatocellular carcinoma cells in an ERK1/2-dependent mechanism [35]. Consistent with these observations, we found that ERK1/2 and PI3K/AKT pathways mediated the effects of EGF and EGFR signals on the expression and activation of MMP-9 in GBM.

Our results also indicated that ERK1/2 and PI3K/AKT pathways result in phosphorylation of STAT3 and STAT5 in GBM. Other authors have implicated STAT3 and ERK signaling pathways in the invasion of HTR8/SVneo cells (an immortalized human trophoblast line) via induction of MMP-9 [36]. However, the scope of that study did not extend to the putative relationship between ERK1/2 + PI3K/AKT and STAT3 + STAT5. We exposed GBM cells to U0126 and LY294002 and found decreased levels of phosphorylated STAT3 and STAT5; these components are responsible for MMP-9 synthesis. Other investigators determined that the JAK/STAT inhibitor ruxolitinib decreases MMP-9 activity in murine and human neutrophils [37]. MMP-9 also was stimulated via JAK-STAT pathways in cigarette smoke-stimulated aortic vascular smooth cells [38]. We extended these findings to include STAT3 and STAT5 in the regulation of MMP-9 expression and activity.

We did not identify binding sites for STAT3 and STAT5 on the *MMP-9* promoter, indicating that MMP-9 is not transcriptionally regulated by these factors in EGF-treated A172 cells. However, STAT3 and STAT5 regulated the phosphorylation of NF-κB, which is responsible for the synthesis of MMP-9. Others have found that STAT3 activates NF-κB in chronic lymphocytic leukemia cells [39]. In addition, interactions between STAT3 and NF-κB can regulate the expression of various genes, including haptoglobin [40]. STAT5 also can stimulate the activity of the NF-κB p65 subunit [41, 42]. Therefore, NF-κB might control the synthesis of MMP-9. In tumors, the NF-κB pathway is essential for the synthesis of MMP-9 [43, 44].

Herein, we elucidated the pathway by which EGFR mediates the stimulatory effects of EGF on the expression and activity of MMP-9 in GBM. EGF stimulates the activities of ERK1/2, PI3K/AKT, STAT3, STAT5, and NF-κB in an EGFR-dependent mechanism, thereby augmenting MMP-9 expression. In turn, activated MMP-9 facilitates abnormal cell proliferation, migration, and invasion, which accelerate the metastasis of GBM.

## MATERIALS AND METHODS

### Reagents

EGF and its inhibitors (LY294002 and U0126) were obtained from Sigma-Aldrich Corp (St. Louis, MO). Quinazoline (QNZ) was purchased from Enzo Life International Inc. (Plymouth Meeting, PA). EGFR, STAT3, STAT5α, STAT5β, p65, and scramble small interfering (si) RNA were obtained from Santa Cruz Biotechnology (Santa Cruz, CA). Complementary DNA (cDNA) plasmids containing MMP-9 were purchased from Origene Technologies (Rockville, MD) and were subcloned to the pCMV6-XL vector. Antibodies against β-actin, MMP-9, MMP-11, EGF, EGFR, phosphorylated (p)-ERK1/2, ERK1/2, p-AKT, AKT, p-STAT3, STAT3, p-STAT5, and STAT5 were purchased from Cell Signaling Technology, Inc. (Danvers, MA). All reagents for quantitative reverse transcription polymerase chain reaction (qRT-PCR) and sodium dodecyl sulfate-polyacrylamide gel electrophoresis (SDS-PAGE) were purchased from Bio-Rad Laboratories (Hercules, CA, USA). All other reagents were obtained from Invitrogen (Carlsbad, CA), unless otherwise specified.

### Cell culture, transfection, and inhibition

GBM A172 cells were maintained in Dulbecco’s Modified Eagle’s Medium (DMEM) supplemented with 10% fetal bovine serum (FBS). MMP-9 expression constructs were introduced into cells with Lipofectamine; control, STAT3, STAT5α, STAT5β, and EGFR siRNAs were introduced into cells with Lipofectamine 2000. A172 cells stably expressing the empty vector or the vector containing MMP-9 cDNA were selected by supplementing the DMEM with G418. Where indicated, cells were treated with EGF in the absence or presence of LY294002, U0126, or QNZ.

### RNA isolation and qRT-PCR

Total RNA was prepared by means of a NucleoSpin RNA Clean-up kit (Macherey Nagel, Bethlehem, PA, USA), and RT was carried out with PrimeScript Reverse Transcriptase (Takara, Dalian, China). Analyses of qRT-PCR were performed with Sybr Green I and a Bio-Rad IQ5 detection system. GAPDH was run in parallel as a reference gene for data normalization. Forward (F) and reverse (R) primers were designed with Primer3 and included MMP-9 (NM_004994) F-GCAGACATCGTCATCCAGTTTG, R-TGCGTTTCCAAACCGAGTTG; EGF (NM_001178130) F-TGGTTCAAAACGCCGAAGAC, R-AACACCAAGCAGTTCCAAGC; EGFR (NM_005228) F-AGGTGAAAACAGCTGCAAGG, R-TTGCACTTGTCCACGCATTC; and GAPDH (NM_002046) F-AAAATCAAGTGGGGCGATGC, R-GGCATTGCTGATGATCTTGAGG.

### Immunoblotting

Cells or tissues were lysed with RIPA buffer. Extracts were resolved by SDS-PAGE and were transferred onto polyvinylidene difluoride membranes. The membranes were incubated with primary antibodies diluted in tris-buffered saline with Tween-20 (TBST). After washing the membranes with TBST 5 min, 3 times, horseradish peroxidase-conjugated secondary antibodies then were applied to detect the primary antibodies. Immunoblots were developed by exposure to an enhanced chemiluminescence reagent (Millipore, Shanghai, China).

### Zymography

Conditioned medium was collected from cell cultures and was mixed with a nonreducing sample buffer. The sample was resolved with 10% SDS-PAGE, and the gel was washed for 1 h at room temperature in a 2.5% (v/v) solution of Triton X-100 to remove the SDS. The gel was transferred to zymogram developing solution (KOMABIOTECH, Seoul, Korea) and was incubated for 72 h at 37°C. The gel then was stained for 30 min with 0.1% (w/v) Coomassie brilliant blue in a solution of 50% (v/v) methanol and 10% (v/v) acetic acid. Destaining was carried out in a solution of 20% (v/v) methanol and 10% (v/v) acetic acid. Areas of lysis were observed as white bands against a black background (Bio-Rad Laboratories, Hercules, CA, USA).

### Promoter assay

A promoter assay was performed, and primers for the truncated *MMP-9* promoter were designed as described previously [23]. Firefly and *Renilla* luciferase activities were determined by means of a dual-luciferase reporter assay kit (Promega, Madison, WI). Standard site-directed mutagenesis was performed to insert point mutations within the binding sites of various transcription factors across the approximately 2000-bp construct. For NF-κB, the following mutation constructs were prepared: mNF-κB, 5′-GGTTTTTCCC-3′ to 5′-GGATTTTCCA-3′. For activator protein 1 (AP-1), the following mutation constructs were made: mAP-1, 5′-CATGGCTCAT-3′ to 5′-CATTGCTTAT-3′; mAP-1, 5′-TTGAGTCAGAA-3′ to 5′-TTGCGTTAGAA-3′. All construct sequences were confirmed by DNA sequencing. Firefly luciferase activities were normalized to *Renilla* luciferase controls. Data were expressed as ratios of EGF to vehicle-normalized firefly luciferase activity, unless stated otherwise.

### ChIP-qPCR

Chromatin immunoprecipitation (ChIP) was performed as described previously [45]. Immunoprecipitated DNA was purified after phenol extraction and was used for qPCR (Upstate Biosystems, NY, USA). Forward and reverse primers for amplification of the *MMP-9* promoter (252 bp) by qPCR were applied as follows: 5′-AAATTTAGCCAGGCGTGGTG-3′ and 5′-ATCGGGCAGGGTCTATATTCAC-3′, respectively.

### Soft agar colony-formation assays

Cells were resuspended in DMEM containing 0.3% agarose and were plated on top of a layer of growth medium with 0.6% agarose in a 6-well plate. Growth medium and inhibitors were replenished every fourth day. Ten days after plating, the colonies were counted and analyzed.

### Proliferation curve

1×10^3^ cells were seeded in serum-free DMEM in a 96-well plate. At specific time points, 10 μl of WST (WST cell proliferation kit, Beyotime, Shanghai, China) was added to each well, and cells were incubated for 3 h. Optical densities at 490 nm were determined, and a proliferation curve was plotted.

### Migration and invasion assay

1×10^5^ cells were seeded into the upper chamber of a 24-well Transwell insert with an 8-μm pore size (Becton Dickinson, Bedford, MA). The lower chamber contained complete DMEM with 10% FBS. After 48 h, cells on the upper side of the chamber were removed with a cotton swab, and the membrane was fixed with 3.7% formaldehyde. Membranes then were stained with 0.1% crystal violet in phosphate-buffered saline (PBS) and were visualized under low magnification (×100). Five micrographic fields (up, down, left, right, center) were analyzed for each membrane. The number of cells in each field that had migrated through the polycarbonate membrane was determined. To evaluate cell invasion, the polycarbonate membrane of the Transwell insert was precoated with Matrigel (Becton Dickinson) before cells were seeded in the upper chamber. The invasion protocol was otherwise identical to the migration protocol described above.

### Immunohistochemistry

Immunohistochemistry staining was performed as described previously [11]. Human GBM tissue was sectioned with a cryostat (10-μm thickness). Sections were mounted to slides, rehydrated in a graded series of ethanol, and submerged in 3% hydrogen peroxide to eliminate endogenous peroxidase activity. The level of MMP-9 then was determined with an immunohistochemical staining kit, according to the manufacturer’s instructions (Invitrogen).

### Tumor growth and metastasis assay

The authors (S.I. or T.V.) injected A172 cells transfected with MMP-9 cDNA or empty vector into NOD/SCID/IL2 receptor gamma knockout mice. Injections were made subcutaneously or via the tail vein. At specific time points, the mice were evaluated for tumor diameter, body weight, and death rate.

### Ethics approval

All animals were handled in accordance with guidelines set forth in the Care and Use of Medical Laboratory Animals (Ministry of Health, People’s Republic of China, 1998), and all experimental protocols were approved by the Laboratory Ethics Committees of China Medical University (Shenyang, China).

### Statistical analysis

Data are expressed as the mean ± standard error (SE) of at least 3 independent experiments. The statistical significance of differences between the means was ascertained with Student’s *t* test or one-way analysis of variance (ANOVA). When the means were found to be significantly different, multiple pairwise comparisons were performed using Tukey’s test.
